# Females fall more from heights but males survive less among a geriatric population: insights from an American level 1 trauma center

**DOI:** 10.1186/s12877-019-1252-6

**Published:** 2019-08-29

**Authors:** Ayman El-Menyar, Elizabeth Tilley, Hassan Al-Thani, Rifat Latifi

**Affiliations:** 1 Clinical Research Trauma and Vascular Surgery, Hamad Medical Corporation and Clinical Medicine, Weill Cornell Medical College, Doha, Qatar; 2 Department of Surgery, Westchester Medical Center and New York Medical College, Valhalla, NY USA; 3 Trauma and Vascular Surgery, Hamad Medical Corporation, Doha, Qatar

**Keywords:** Geriatrics, Trauma in elderly, Mechanism of injury, Injury severity, Fall from height

## Abstract

**Background:**

Approximately one third of subjects ≥65 year old and half of subjects ≥80 years old sustain a fall injury each year. We aimed to study the outcomes of fall from a height (FFH) among older adults. We hypothesized that in an elderly population, fall-related injury and mortality are the same in both genders.

**Methods:**

A retrospective analysis was conducted between January 2012 and December 2016 in patients who sustained fall injury at age of at least 60 years and were admitted into a Level 1 Trauma center. Patients were divided into 3 groups: Gp-I: 60–69, Gp-II: 70–79 and Gp-III: ≥80 years old. Data were analyzed and compared using Chi-square, one-way analysis of variance (ANOVA) and logistic regression analysis tests.

**Results:**

Forty-three percent **(**3665/8528) of adult trauma patients had FFH and 59.5% (2181) were ≥ 60 years old and 52% were women. The risk of fall increased with age with an Odd ratio (OR) 1.52 for age 70–79 and an OR 3.40 for ≥80.

Females fell 1.2 times more (age-adjusted OR 1.24; 95% CI 1.05–1.45) and 47% of ≥80 years old suffered FFH. Two-thirds of FFH occurred at a height ≤ 1 m. Injury severity (ISS, NISS and GCS) were worse in Gp-II, lower extremities max Abbreviated Injury score (max AIS) was higher in Gp-III. Overall mortality was 8.7% (Gp-I 3.6% vs. 11.3% in Gp-II and 14% in Gp- III). Males showed higher mortality than females in the entire age groups (Gp-I: 4.6% vs 1%, Gp-II: 12.9% vs 4.2% and Gp-III: 17.3% vs 6.9% respectively). On multivariate analysis, shock index (OR 3.80; 95% CI 1.27–11.33) and male gender (OR 2.70; 95% CI 1.69–4.16) were independent predictors of mortality.

**Conclusions:**

Fall from a height is more common in older adult female patients, but male patients have worse outcomes. Preventive measures for falls at home still are needed for the older adults of both genders.

**Electronic supplementary material:**

The online version of this article (10.1186/s12877-019-1252-6) contains supplementary material, which is available to authorized users.

## Background

Adults age 60 and older are the fastest growing segment of the population in the world. In the USA the number of the older adults is expected to reach 89 million by 2050, while globally from 2025 to 2050, the older population is projected to reach 1.6 billion. Each year, 3 million elderly people are treated in the emergency department for fall-related injuries which are always associated with catastrophic functional outcomes [1–3]. Over 800,000 patients per year are hospitalized for a fall related injury, most commonly, head injuries or hip fractures [1]. Falls are one of the most common causes of traumatic brain injuries [2]. In the year of 2015, the total medical costs for fall-related injuries accounted for more than $50 billion [3]. Falls are the leading cause of injury and death among the older adults [1–3]. Approximately every third person (30%) of the population over the age of 65 and every other one (50%) over the age of 80 sustain falls each year [4, 5]. Recent estimates indicated that the incidence of falls leading to emergency admissions and mortality have been increasing over the past few years [6–9].

It has been estimated that one in four of people in the Western countries will be at geriatric age by the year 2030, and health care costs for fall-related injuries will be a major issue in the geriatric population [10, 11].

Morbidity and mortality are more common in older adults trauma patients compared to younger patients [12, 13]. Age is an independent predictor of survival following trauma [14]. In addition, older adults trauma patients have distinct patterns of injuries because of their unique physiologic, behavioral, and anatomical characteristics, thus, trauma is expected to dramatically affect the older adults quality of life [15].

Several prediction scores are used to manage trauma patients, including Injury Severity Score (ISS), Trauma Injury Severity Score (TRISS), Revised Trauma Score (RTS), New Injury Severity Score (NISS) and Shock Index (SI) [16, 17].

Although our trauma center draws patients from many other counties in New York state (over 3.5 million population), the largest portion come from Westchester county, which is just north of New York City, and had a population estimate of approximately 980,000 in 2017, according to U.S. Census [16]. The county covers an area of 450 mile^2^ and includes six cities, 19 towns, and 23 villages [16]. The county is racially diverse with an estimate of 73.5% (White alone) and 24.9% (Hispanic or Latino). Twenty-two percent of the population is under the age of 18 and 16.6% is aged 65 years or older and 51.5% of the population is female gender [16].

There is limited information available about clinical characteristics and outcomes of fall related injuries among the geriatric populations in the state of New York (NY) and its counties. The purpose of this study is to find out clinical characteristics, patterns, and outcomes of fall related injuries in older adults patients admitted to a Level I regional trauma center. We hypothesized that in older adults, fall-related injury and mortality are the same in both genders.

## Methods

This retrospective study was conducted at the Westchester Medical Center, Valhalla, NY, a 895 bed facility and a Level I regional trauma center that provides care to > 3.5 million people in New York’s Hudson Valley region and beyond. The medical center covers a population across 6200 mile^2^.

We retrieved data from the Trauma Registry System of our institution for all adult trauma patients ≥18 years old between January 2012 and December 2016. The registry follows the American College of Surgeons guidelines for data acquisition and storage in a Level I trauma center registry. This study follows STROBE checklist (Additional file 1: Table S1). Data were analyzed and compared on an annual basis across a 6-year period. Inclusion criteria included all men and women who sustained fall from height at age 60 years old and above. We excluded patients who died at the scene or lack relevant information (i.e., other mechanism of injury or incomplete fall-related data). Patients were divided into 3 groups based on their age: Group-I: 60–69, Group-II: 70–79 and Group-III: ≥80 years old.

Variables used in the data analysis for this study included demographics, age, gender, mechanism of injury (MOI), vital signs on arrival, the first Glasgow Coma Scale (GCS) score in the emergency department, height of fall (< 1 m, 1-3 m, 3-6 m, > 6 m), alcohol use, Abbreviated Injury Scale (AIS) scores for each body region, ISS, New Injury Severity Score (NISS), Trauma and injury severity score (TRISS), blood transfusion, length of hospital stay (HLOS), in-hospital mortality, and diabetes mellitus and alcoholism. In brief, AIS, ISS, and NISS are anatomical, GCS is a physiological scoring system, and TRISS is a combined scoring system for survival prediction.

Definition of fall from height: A fall is defined as an injury to a person that occurs after landing on the ground after falling from a higher place or fall from any distance that causes injury and requires a transfer to the emergency department (< 1, 1–3, 3–6, > 6 m(m)). Shock index (SI) was defined as admission heart rate divided by the systolic blood pressure [18, 19]. Normal SI range is between 0.50–0.70. The ISS scores range from 1 to 75, being one the least severe and 75 the most severe trauma injury. Any injury coded AIS 6 implies an ISS of 75. The AIS code for each injury ranges from 1 (minor injuries) to 6 (maximum injuries, almost always fatal). The NISS results from the sum of the squares of the three highest AIS scores regardless of the body region affected. TRISS (probability of survival) is a combination index based on Trauma Score (RTS), (ISS), and patient’s age. The higher the TRISS value, the better the patient survival.

This study granted ethical approval from the Westchester Medical Center, and New York Medical College, Valhalla, NY, USA (IRB approval number, L-12,432).

### Statistical analysis

Descriptive and inferential statistics were applied for data analysis. Data were presented as mean (standard deviation), median (range), median (interquartile range; IQR) or number (%) as appropriate. Data were analyzed and compared using Chi-square and one-way analysis of variance (ANOVA) tests. The Post Hoc Bonferroni test was used for the pairwise comparison. We compared patients’ characteristics and outcomes in each gender, age groups (60–69, 70–79 vs ≥80 years) and within each gender (< vs > 65 years old) and different heights. The association between age, gender and falls was assessed by logistic regression. The association between the fall height and other variables were assessed by ANOVA. For predictors of mortality among patients with fall-related injury, multivariable logistic regression analysis was conducted after adjustment for the potential relevant variables and significant univariate differences. . Data were expressed with odds ratio (OR) and 95% confidence intervals (CI). Statistical significance was defined as a *p*-value < 0.05. Data were analyzed using Statistical Package for the Social Sciences (SPSS) for Windows Version 21.0 (SPSS Inc.; Chicago, IL, USA).

## Results

Out of 8528 adult trauma patients, 3665 (43%) had fall related injuries, of them 2181 (59.5%) were ≥ 60 years old and 1132 (52%) were females. While the risk of fall increased with age with an OR 1.52 (95% CI 1.25–1.84) for patients between ages 70 and 79, the probability of fall was 3.4 times greater for patients’ age ≥ 80 (an OR 3.40; 95% CI 2.76–4.08). Female gender was associated with increased risk of fall by 1.2 times (age-adjusted OR 1.24; 95% CI 1.05–1.45).

Table 1 shows the demographics, characteristics and outcomes in patients with fall-related injury based on the age group. There were no significant differences for the proportions of falls across the years among age groups; however, almost half of older adults (47%) sustained fall-related injury at age ≥ 80 years old. Whites were the most common to experience falls in all age groups. The majority of older adults sustained a home related fall (72.9%).

**Table 1:** Demographics, characteristics and outcomes based on age group in fall-related injured patients

Variable	Age 60–69	Age 70–79	Age ≥ 80	*P*
Total (*N* = 2181)	584(27%)	570(26%)	1027(47%)	
White (*N* = 1826)	450 (77.1%)	456 (80.0%)	920 (89.6%)	0.001
Black (*N* = 93)	43 (7.4%)	28 (4.9%)	22 (2.1%)	
Hispanic (*N* = 116)	48 (8.4%)	29 (5.1%)	39 (3.8%)	
Alcohol consumption (N*** = 231, 10.6%)	38 (6.5%)	34 (6.0%)	43 (4.2%)	0.001
NISS*	14.43, 12.0 (1–75)	15.93, 12.0 (1–75)	14.96, 12.0 (1–75)	0.001
ISS*	10.82, 9.0, (1–75)	11.99, 9.0, (1–75)	11.73, 9.0, (1–50)	0.001
Shock index**	0.62 ± 0.16	0.61 ± 0.19	0.60 ± 0.16	0.001
TRISS**	0.9258 ± 0.139	0.9085 ± 0.162	0.920 ± 0.141	0.001
Year				
2012 (*N* = 438, 20.1%)	114 (19.5%)	127 (22.3%)	197 (19.2%)	0.372
2013 (*N* = 448, 20.5%)	119 (20.4%)	115 (20.2%)	214 (20.8%)	
2014 (*N* = 430, 19.7%)	125 (21.4%)	114 (20.0%)	191 (18.6%)	
2015 (*N* = 417, 19.1%)	115 (19.7%)	97 (17.0%)	205 (20.0%)	
2016 (N = 448, 20.5%)	111 (19.0%)	117 (20.5%)	220 (21.4%)	
Place of trauma				
Public (*N* = 156)(27.1%)	32 (33.3%)	15 (14.9%)	16 (8.05)	0.001
Residence (*N* = 420) (72.9%)	64 (66.7%)	86 (85.1%)	185 (92.0%)	
Fall Height in meter*	8.67, 8.0, (1–40)	7.69, 6.0, (1–30)	5.45, 3.0, (1–40)	0.001
Glasgow coma scale				
Mild (> 12),*N* = 1957	531 (91.2%)	506 (88.9%)	920 (90.1%)	0.243
Moderate (8–12),*N* = 76	17 (2.9%)	19 (3.3%)	40 (3.9%)	
Severe (< 8), *N* = 139	34 (5.8%)	44 (7.7%)	61 (6.0%)	
Max AIS**				
Head	3.03 (1.18)	3.2 (1.22)	3.06 (1.25)	0.199
Upper extremities	1.89 (.56)	1.87 (.54)	1.77 (.57)	0.040
Lower extremities	2.16 (.65)	2.34 (.75)	2.46 (.75)	0.001
Spine	2.39 (.80)	2.5 (.77)	2.4 (.64)	0.508
Diabetes mellitus (*N* = 536)(25%)	141 (24.1%)	172 (30.2%)	223 (21.7%)	0.001
Rib injury (*N* = 369) (16.9%)	104 (17.8%)	88 (15.4%)	177 17.2%)	0.001
Blood Transfusion (*N* = 321) (6.3%)	71 (13.5%)	70 (13.6%)	180 (19.3%)	0.003
Hospital stay, days*	8.10, 5.0, (1–95)	9.8, 6.0, (1–302)	7.95, 6.0, (1–96)	0.001
Mortality (*N* = 28 (8.7%)	5 (3.6%)	11 (11.3%)	12 (14%)	0.001

* = mean, median and range. ** = mean and standard deviation, ***N = number of patients

**Fig. 1:**
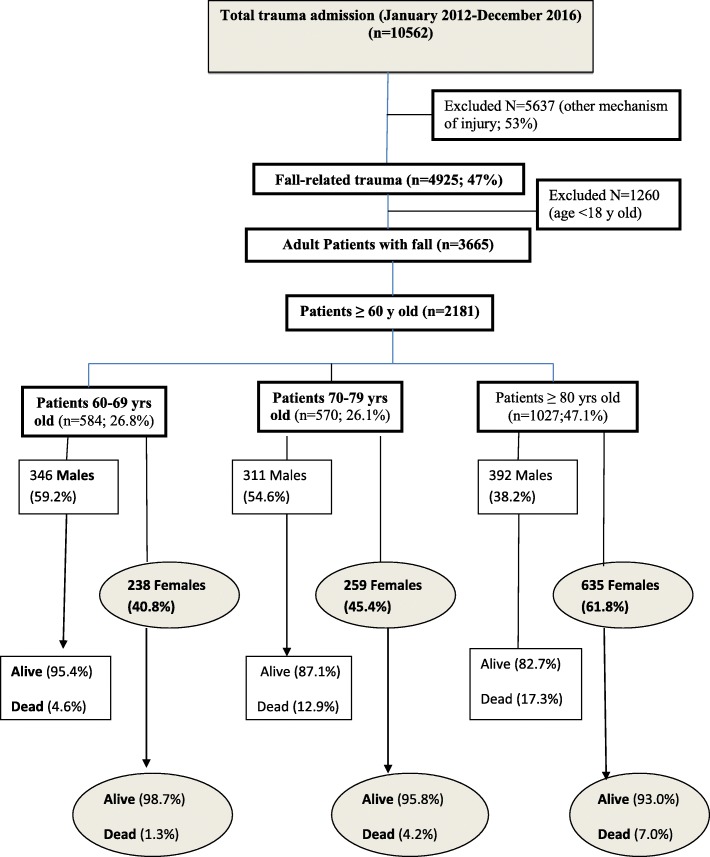
Study design and breakdown of Fall-related injury and mortality in each age group and gender

Injury severity in terms of ISS, NISS, TRISS and GCS were worse in group II in comparison to group I and III (*p* < 0.001). Shock index was greater in group 1 in comparison to the other groups (*p* < 0.001). Lower extremities max AIS was higher in group III (*p* < 0.001) whereas upper extremities max AIS and rib fractures were greater in group I (*p* < 0.001). Head and spine injuries were comparable among all the groups. Alcohol consumption and diabetes mellitus were reported in 11 and 25% of the study cohort, respectively. Alcohol consumption was more frequent in group I (6.5%) and II (6%) whereas diabetes mellitus was more prevalent in group II (30.2%) and group I (24.1%). Blood transfusion was required in 6.3% of the cohort; particularly in group III (19.3%) in comparison to the other groups (*p* < 0.001). The mean hospital length of stay was greater in group II (*p* < 0.001). In Table 2, patients with fall-related injury were stratified according to the height of fall. The proportions of fall from a height less than 3 m were more frequent in females whereas fall from 3 m or higher was more in males. Two-thirds of fall-related injury occurred at a height ≤ 1 m. Although statistically it was not significant, the mortality was higher in those who fell down from < 1 m (8.8%) and those who fell down from > 6 m distance (8.7%) in comparison to other heights. Figure 1 shows the study design and breakdown of Fall-related injury population. It also shows the proportion of falls and mortality in each age group. Overall mortality was 8.7% (group I 3.6, 11.3, and 14% in group I-III, respectively). Mortality was significantly higher among males in comparison to females in all the age groups (group I: 4.6% vs 1%, group II: 12.9% vs 4.2% and group III: 17.3% vs 6.9% respectively) (*p* < 0.003). Table 3 shows differences between older adults females and males. The mortality was 2-fold higher in males. Males had higher fall heights, ISS and NISS, fewer GCS, higher proportion of rib fractures, diabetes and smoking in comparison to females. A subanalysis within each gender, shows patients’ characteristics and outcomes based on age < 65 years vs > 65 years old (Table 4). It shows that in females, the fall was more frequent in those who were of age 65 and above whereas in males the majority of fall was in the younger age. However, the severity of injury, rib fractures, diabetes mellitus, blood transfusion and mortality were more frequent in males aged 65 and above. Of note, females were 4 years younger than males in those who in the age group of < 65 whereas females were 3 years older than males in those who in the age group of 65 and above. Additional file 2: Table S2 shows fall-related injury and mortality by year (2012–2016). Fall-related injury in WCMC ≥60 years was stationary from 2012 to 2016 ranging between 19 and 20.5%. Fall-related mortality in WCMC-Females ≥60 years dropped from 7.5% (2012) to 1.2% (2016). Fall-related mortality-WCMC-Males ≥60 years ranged between 11.1 and 14.4% across the 6 years.

**Table 2:** patients characteristics based on the height of fall (*n* = 2181)

Variable	< 1 m (*n* = 1438; 66%)	1-3 m (*n* = 292, 13%)	> 3-6 m (*n* = 130, 6%)	> 6 m (*n* = 321, 15%)	*P*
Female *N*(%)	811 (56.4)	171 (58.6)	58 (44.6)	92 (28.7)	0.001
Male *N*(%)	627 (43.6)	121 (41.4)	72 (55.4)	229 (71.3)	0.001
Age (years) **	79(60–103)	81(60–102)*	74 (60–99)*	71 (60–98)*	0.001
ISS**	10 (9.7–10.3)	10.7 (9.9–11.5)*	12.1 (10.9–13.3)*	13.4 (12.8–14.1)*	0.001
NISS**	13.2 (12.8–13.7)	13.7 (12.7–14.8)*	16.7 (15.1–18.3)*	17.8 (16.9–18.7)*	0.001
Abdomen AIS**	2.4 (2.2–2.6)	2.1 (1.7–2.5)	2.6 (2–3.2)	2.2 (1.9–2.4)*	0.235
Head AIS**	3.2 (3.1–3.3)	3.1 (2.9–3.3)	2.9 (2.7–3.1)	2.8 (2.7–3)*	0.001
Thorax AIS**	2.7 (2.6–2.8)	2.6 (2.4–2.9)	2.7 (2.5–3)	2.7 (2.6–2.8)	0.968
Upper extremity AIS**	1.8 (1.8–1.9)	1.9 (1.7–2)	2 (1.8–2.1)	1.9 (1.9–2)	0.044
Spine AIS**	2.5 (2.4–2.6)	2.5 (2.3–2.6)	2.5 (2.2–2.7)	2.3 (2.3–2.4)*	0.013
Lower extremity AIS**	2.4 (2.3–2.4)	2.5 (2.4–2.6)	2 (1.9–2.2)*	2 (1.9–2.1)*	0.001
Probability of survival (TRISS) **	0.94 (0.93–0.94)	0.94 (0.92–0.95)	0.93 (0.91–0.95)	0.93 (0.92–0.94)	0.069
Hospital stay, days	7.7 (7.2–8.1)	7.9 (7–8.8)	7.8 (6.6–9)	8.1 (7.5–8.7)	0.800
Mortality	127 (8.8%)	18 (6.2%)	9 (6.9%)	28 (8.7%)	0.44

* Post hoc Bonferroni test significant (*p* < 0.05) and reference group is < 1 m height of fall,** = median, range. AIS = abbreviated injury score, ISS: injury severity score, NISS = new ISS, TRISS = Trauma and injury severity score

**Table 3:** comparison between females and males aged > 60 years old

Variable	Females	Males	*P*
Person aged > 80 years	4.8%	7.2%	0.37
NISS score	13.5 ± 10	16.7 ± 12.6	0.001
ISS category			
16–24	16%	20%	0.001for all
> 24	6%	13%	
GCS category			
8–12	2.6%	4.5%	0.002 for all
< 8	4.7%	8.4%	
Max AIS head > 2	67%	73%	0.09
Alcohol consumption (% of persons)	4.2%	6.2%	0.02
Smoking	4%	8.7%	0.001
Diabetes mellitus	22.8%	26.4%	0.05
Rib fractures	13%	21%	0.001
Fall heights median (IQR)	< 1(< 1–3)	3(< 1–6)	0.02
Mortality	5.2%	12.4%	0.001

IQR = interquartile range

**Table 4:** patients characteristics and outcomes within each gender based on the age groups (< vs > 65 years)

Variable	Females (*n* = 1510; 41.2%)			Males (*n* = 2155; 58.8%)		
	< 65 yrs	> 65 yrs	*p*	< 65 yrs	> 65 yrs	*p*
Patients number	488(32.3%)	1022(67.7%)	0.001	1300(60.3%)	855(39.7%)	0.001
Mean age ± SD	27 ± 22	81 ± 9.0	0.001	31 ± 20	78 ± 8.0	0.001
ISS^a^ score	5(4–9)	9(5–14)	0.001	9(4–13)	10(8–17)	0.001
NISS^a^ score	8(4–12)	10(9–17)	0.001	9(4–17)	13(9–22)	0.001
TRISS^a^ score	0.994(0.983–0.996)	0.968(0.943–0.975)	0.001	0.994(0.983–0.996)	0.965(0.933–0.970)	0.001
Fall height^a^ in meter	4(3–9.5)	3(3–8)	0.004	8(4–15)	6(3–11)	0.001
Diabetes mellitus	6.3%	23.3%	0.001	6.9%	26.8%	0.001
Rib fractures	4.2%	13.5%	0.001	11%	20%	0.001
GCS ≤ 8	4.3%	5.6%	0.17	5.7%	8.9%	0.002
Person received blood transfusion	4.1%	15.6%	0.001	5.2%	18.1%	0.001
Mortality *N*;(%)	8(1.6%)	56(5.5%)	0.001	32(2.5%)	116(13.6%)	0.001

^a^Data presented as median (interquartile range)

Multivariable logistic regression analysis (Table 5) showed that SI (OR 3.80;95% CI 1.27–11.33), male gender (OR 2.70; 95% CI 1.69–4.16) and GCS (OR 0.814; 95% CI 0.772–0.858) were independent predictors of mortality among elder patients who fell down after adjustment for age, diabetes mellitus, ISS, height of fall and need for blood transfusion.

**Table 5:** predictors of mortality in patients with fall-related injury

Variable	Odd ratio	95% CI		*P*
Age	1.059	1.035	1.085	0.001
Fall height	0.924	0.877	0.973	0.003
Injury Severity Score	1.123	1.094	1.153	0.001
Sex (male)	2.70	1.69	4.16	0.001
Diabetes mellitus	1.253	0.769	2.041	0.366
Blood Transfusion	1.455	0.915	2.314	0.113
Glasgow Coma scale	0.814	0.772	0.858	0.001
Shock Index	3.796	1.271	11.331	0.017

## Discussion

This study analyzes the demographics and clinical characteristics of fall related injuries observed in male and female geriatric population presented to a Level I trauma center in Westchester County, New York. This is a unique study with a large sample size that emphasizes the impact of gender on the incidence and outcome of fall from height among older adults.

This study shows that fall-related injuries represent 43% of the total trauma admissions and nearly 60% of them are of age 60 and above. Almost half of older adults sustained fall-related injury at age ≥ 80 years old. Approximately three-quarter of older adults sustained a home related fall. Across the study period from 2012 to 2016, there were no significant changes in the proportions of falls among different age groups; however, mortality decreased from 10 to 5.6%. This drop in mortality could be in part explained by the higher mean ISS in 2012 (13 ± 8) in comparison to 10 ± 7.6 in 2016; in addition to the improvement in the health care system. Of note, mortality in females decreased from 7.5% in 2012 to 1.2% in 2016 (*p* = 0.02), whereas it varied in males from 12.4% in 2012 to 11.1 in 2016 with a peak in 2014 (14.4%). The mean age, ISS and GCS did not show significant changes per year in female gender and therefore it could not explain the drop in mortality between 2012 and 2016. The risk of fall increases significantly with the increase in age with an OR of 3.4 in patients aged 80 and above. Furthermore, our study shows that female gender is associated with increased risk of fall regardless of the age. The mortality was greater in males by 2 times, which could be related to the higher ISS, head AIS, height of fall and NISS and lower GCS in addition to the greater frequency of having DM, alcohol consumption, smoking habit, and rib fractures.

Prior data reported that almost one third of the older adults population fall at least once per year [20–24], however, our study reported one fifth of older adults falls once per year.

Our data shows that women between 60 and 79 years old sustain more fall injury, however, at age of 80 and above, men fell more than women. Prior reports conducted on emergency department visits showed that women experienced fall-related injury more than men [17, 25–28]. In contrary, two studies reported that men comprised a more proportion of fall-related emergency visits [29, 30]. Recently, Galet et al. [24] evaluated WHO mortality and readmission database between 2010 and 2014 among older adults. The authors observed 1.4 and 2% increase in the fall-related mortality and readmission for a subsequent fall, respectively. Fall was the most common mechanism of injuries in women who required hospitalization and the majority of fall patients readmitted within 30 days were also women [24].

It is difficult to establish the factors affecting mortality in falls [23]. The WHO database showed that older adults with uncomplicated and complicated diabetes mellitus were at 16 and 31% increased risk of being admitted for fall injury, respectively [24]. Also it was observed that anemic patients had a 21% increased risk of being admitted for a fall injury. Alcohol consumption and diabetes mellitus were reported in 11 and 25% of our study cohort, respectively.

In the present study, SI and male gender were independent predictors of mortality among elder patients who fell down after adjustment for age, diabetes mellitus, GCS, ISS, height of fall and need for blood transfusion. Pandit et al. [31] studied 217,190 geriatric trauma patients and found that SI is an accurate and specific predictor of significant bleeding and mortality in geriatric trauma patients. Higher SI after trauma in older adults prerequisites transfer to a Level 1 trauma center [31].

In 2010, Bennett et al. [32] studied the outcomes in 422 patients ≥80 years old vs 898 patients aged 60–79 years old after traumatic injury. The first group had significantly higher risk-adjusted in-hospital mortality (OR 1.94; 95% CI 1.14, 3.31). In our study this elderly group who sustained fall injury after adjustment for gender and GCS had a higher mortality with OR 2.871;95% CI 1.97–4.19).

In the United States, falls from the standing height are considered the second cause of death due to unintentional injuries in the older adults [10]. Based on the present study; home related falls were the most common falls in older adults. In 2011 Rapp et al. [33] analyzed nearly 70,000 elderly for falls that occurred in residences in Germany and observed a higher frequency of falls among male patients, and approximately 75% of the events occurred in the bedroom or bathroom. A previous study with a small sample size showed that elderly who sustained falls-related injury at bathroom were significantly associated with the female gender and high mortality rate [34].

### Limitations

The limitations of this study include the use of a retrospective design and the lack of availability of data regarding the risk factors, other than diabetes and alcoholism, such as pulmonary, cardiac and stroke in addition to the circumstances of the mechanism of injury and readmissions. Although we intended to include all fall-related injuries in older adults, selection bias cannot be ignored. We did not intend to study those who brought in dead (BID) or died at the scene as these data were not available for this analysis. There is a significant number of patients with missing information regarding the location of fall (home, workplace or public). Additionally, our single center study is not a representative of the entire population of United States and our results may therefore not be generalizable. When comparing census data of Westchester County to the U.S. in general, there is a higher percentage of Hispanic or Latino, in Westchester County. The U.S. has an average of 18.1% versus 24.9% in Westchester County. Westchester County is slightly more racially diverse than that of the rest of the United States, on average. Additionally, Westchester has slightly older adults than the average of the United States, where Westchester had 16.6% over aged 65, the U.S. in general has 15.6%. However, we do not have data of county origin of patients. Another limitation of our study is the lack of data on anticoagulants, antihypertensive, hypoglycemic agents and polymedications. Most recently, the frailty index has become a popular tool in predicting outcomes in older adults trauma patients, however it was lacking in our data [35].

## Conclusions

Falls from height among the elderly is a major public health concern, and increases with age, particularly among females. However, males have worse outcomes. Preventive measures for falls at home among older adults should have a priority in the major healthcare action plans.

## Additional files

Additional file 1: **Table S1.** STROBE Statement—Checklist of items that should be included in reports of ***cohort studies***. (DOC 93 kb)

Additional file 2: **Table S2.** Fall-related injury and mortality by year. (DOCX 13 kb)

## Data Availability

Data are available upon request at Westchester Medical Center after getting IRB approval and signed sharing data agreement form.

## Abbreviations

AIS: Abbreviated injury scale
FFH: Fall from height
GCS: Glasgow coma scale
ISS: Injury severity score
NISS: New ISS
TRISS: Trauma ISS
