# Supplementary material for: Females fall more from heights but males survive less among a geriatric population: insights from an American level 1 trauma center
Source: BMC Geriatr. 2019 Aug 29;19:238. doi: 10.1186/s12877-019-1252-6 (PMC6716940; doi:10.1186/s12877-019-1252-6)
Supplement: Supplementary file 2 — Table S2. Fall-related injury and mortality by year. (DOCX 13 kb) [file 12877_2019_1252_MOESM2_ESM.docx]

| **Additional file 2: Table S2: fall-related injury and mortality by year** | | | | | |
| --- | --- | --- | --- | --- | --- |
|  | **2012** | **2013** | **2014** | **2015** | **2016** |
| Fall-related injury in WCMC ≥ 60 yrs | 20% | 20.5% | 19.7% | 19% | 20.5% |
| Fall-related mortality in WCMC | 5.1% | 5.1% | 4.4% | 3.8% | 3.1% |
| Fall-related Mortality WCMC≥ 60 yrs | 10% | 9.6% | 9.3% | 7.2 | 5.6% |
| Fall-related mortality in WCMC-Females ≥60 yrs | 7.5% | 6.5% | 4.8% | 6.3 | 1.2% |
| Fall-related mortality-WCMC-Males ≥ 60 yrs | 12.4% | 12.5% | 14.4% | 8.3% | 11.1% |
| WCMC= patients attending Westchester medical center, Valhalla, NY | | | | | |
